# Threading the Pieces Together: Integrative Perspective on SARS-CoV-2

**DOI:** 10.3390/pathogens9110912

**Published:** 2020-11-04

**Authors:** Akshay Kanakan, Neha Mishra, Janani Srinivasa Vasudevan, Shweta Sahni, Azka Khan, Sachin Sharma, Rajesh Pandey

**Affiliations:** INtegrative GENomics of HOst-PathogEn (INGEN-HOPE) Laboratory, CSIR-Institute of Genomics and Integrative Biology (CSIR-IGIB), Delhi 110007, India; akshay.kanakan@igib.res.in (A.K.); neha18239@iiitd.ac.in (N.M.); jananisv@igib.res.in (J.S.V.); shweta.sahni@igib.res.in (S.S.); azka.khan@igib.res.in (A.K.); sachin.sharma@igib.in (S.S.)

**Keywords:** SARS-CoV-2, host response, co-infection, comorbidity, integrative genomics, drugs, therapeutics, vaccines

## Abstract

The severe acute respiratory syndrome coronavirus 2 (SARS-CoV-2) pandemic has challenged the research community globally to innovate, interact, and integrate findings across hierarchies. Research on SARS-CoV-2 has produced an abundance of data spanning multiple parallels, including clinical data, SARS-CoV-2 genome architecture, host response captured through transcriptome and genetic variants, microbial co-infections (metagenome), and comorbidities. Disease phenotypes in the case of COVID-19 present an intriguing complexity that includes a broad range of symptomatic to asymptomatic individuals, further compounded by a vast heterogeneity within the spectrum of clinical symptoms displayed by the symptomatic individuals. The clinical outcome is further modulated by the presence of comorbid conditions at the point of infection. The COVID-19 pandemic has produced an expansive wealth of literature touching many aspects of SARS-CoV-2 ranging from causal to outcome, predisposition to protective (possible), co-infection to comorbidity, and differential mortality globally. As challenges provide opportunities, the current pandemic’s challenge has underscored the need and opportunity to work for an integrative approach that may be able to thread together the multiple variables. Through this review, we have made an effort towards bringing together information spanning across different domains to facilitate researchers globally in pursuit of their response to SARS-CoV-2.

## 1. Introduction

The outbreak of COVID-19 in late 2019 in China soon transformed into a pandemic with global implications. In March 2020, the World health organization (WHO) declared the disease as a Public Health Emergency of International Concern (PHEIC). The cause of the SARS-like disease was attributed to a new zoonotic beta-coronavirus, named SARS-CoV-2 [1]. Human to human transmission of the virus is highly efficient. The clinical symptoms of the disease are much like other respiratory virus infections, including atypical pneumonia. Radiologically, however, COVID-19 is characterized by multifocal ground-glass opacification of the lungs [2]. Patients diagnosed with SARS-CoV-2 infection present a spectrum of clinical symptoms, typically dry cough and fever, and less commonly myalgia, anosmia, loss of taste, and occasional diarrhea, while severe cases progress to acute respiratory distress syndrome (ARDS) and pneumonia [3,4]. COVID-19 presents contradictions in the sense that while the majority (nearly 80%) of laboratory-confirmed cases remain mild or at most moderately affected, others upon infection progress rapidly to severe disease (nearly 14%) or critical illness, including death [5].

Strategizing and designing the response against the current SARS-CoV-2 pandemic requires an in-depth integrative understanding of hierarchical aspects of both host and pathogen. Thus, the review starts with understanding the virus itself on different levels. It is layered upon with other important modulators like the role of host response and predisposition by existing comorbid conditions. Through this review, we make an effort towards facilitating our knowledge of COVID-19 disease by integrating the available information.

## 2. Epidemiology of COVID-19

First seen as idiopathic pneumonia cases in Hubei Province in Wuhan, China, the disease COVID-19 is caused by the SARS-CoV-2 virus. SARS-CoV-2 is primarily transmitted via aerosols, but there is mounting evidence of its airborne transmission as well, enhancing the possibility of its spread. A recent report has highlighted that SARS-CoV-2 remains viable in aerosols for up to three hours [6]. The incubation period refers to the period from when a person is first infected up to the time when the individual first presents the symptoms of the associated disease. For SARS-CoV-2, the median incubation period is observed to be 5–6 days, although it can range from 1–14 days, with outlier cases showing incubation periods up to 24 days [7]. The serial interval, which is the time a virus takes to transmit from one affected individual to another, is 3.96 days, with a standard deviation of 4.75 days [8,9,10]. Studies report that the SARS-CoV-2 mean basic reproductive number (R_0_) ranged from 2.28 on the Diamond Princess cruise ship [11] up to 5.7 in Wuhan, China [12]. The effective reproductive number (Re) better reflects the viral reproduction with control measures that have been estimated to significantly reduce the calculated R_0_. In Germany, an approximate R_e_ of 1 has been seen since 22 March 2020. Such reductions have also been seen in other European countries following non-pharmaceutical interventions such as social distancing, masks, and sanitization (SMS) [13].

**Figure 1:**
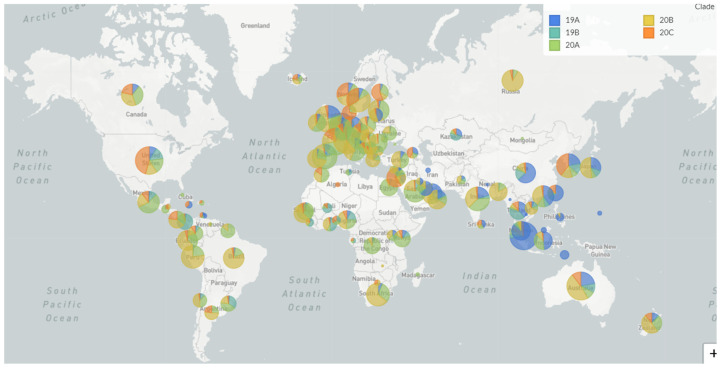
Global distribution of SARS-Cov-2 clades (Nextstrain (https://nextstrain.org/ncov/global?c=clade), updated 3 October 2020).

Nextstrain and the Global Initiative on Sharing All Influenza Data (GISAID https://www.gisaid.org/) are two open-source tools for the collection and visualization of pathogen genome data that help us understand pathogen evolution and epidemic spread (Figure 1). For SARS-CoV-2, 140,502 genomic sequences have been submitted to GISAID (8 October 2020). For tracking the flow of viral strains through the world, certain stable mutations are used to define a clade of the virus which can be identified by genomic surveillance of the virus. Such classification aids in epidemiological studies to quantify the spread of a virus in a population. The existing clades, as defined by Nextstrain, and their global distribution are shown in Table 1.

## 3. The Virus

SARS-CoV-2, a positive-sense, single-stranded, enveloped RNA virus belonging to the Coronaviridae family in the genus *Betacoronavirus*, is the cause of the 2019 pandemic of COVID-19 disease [1,14]. The virus has similar genetic ancestry and belongs to the genus of SARS-CoV that led to the 2003 epidemic and Middle East respiratory syndrome-related coronavirus (MERS-CoV) 2011 pandemic outbreak. The novel virus is closest to its evolutionary relative, bat SARS/SARS-like coronaviruses (RaTG13, ZXC21, and ZC45) [15]. The spike protein structure of SARS-CoV-2 is seen to be much more closely related to pangolin CoV, thus indicating the existence of a secondary host reservoir of SARS-CoV-2 in pangolins (Order: *Pholidota*) [16]. However, these findings await conclusive evidence to establish the pangolin as a candidate for SARS-CoV-2 origin. Homologous recombination, an established evolutionary force behind the emergence of new variants, seems to be the plausible cause for the origin of this novel pathogenic virus [17,18]. A total of 380 amino acid substitutions have been observed in SARS-CoV-2 compared to SARS-like CoVs [19]. The highest level of alteration of ~19% is seen in the spike protein (S), which is essential for viral anchoring and entry into host cells, whereas only one mutation is observed in the RNA-dependent RNA polymerase (*RdRP*) gene of the virus [20].

**Figure 2:**
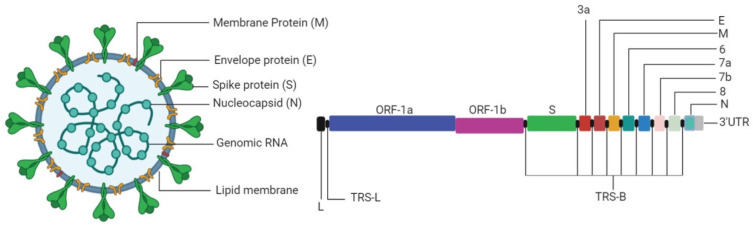
Schematic representation of SARS-CoV-2 viral particle and genomic architecture. The colored region shows the viral genes and the regions in black represent the interspersed regulatory elements.

SARS-CoV-2 has a 30 kb unfragmented genome that replicates continuously but undergoes discontinuous transcription in the host cell. Viral genomes can be divided into three sections that have two open reading frames (ORFs) at the 5′ end, ORF1a and ORF1ab, and 12 ORFs at the 3′ end consisting of four structural genes and eight accessory genes ORFs [9] (Figure 2). ORF1a and ORF1b produce 15 non-structural proteins (nsps) that are co- and post-translationally processed to produce vital proteins such as RdRp. These two ORFs are directly translated from the viral RNA upon entry into the host cell, thus generating pp1a and pp1ab polypeptide by ribosomal switching at the 3′ end of ORF1a. The proteolytic processing of these ORFs happens due to proteases produced by ORF1a, such as nsp3 and nsp5 [21].

### 3.1. Transcription

**Figure 3:**
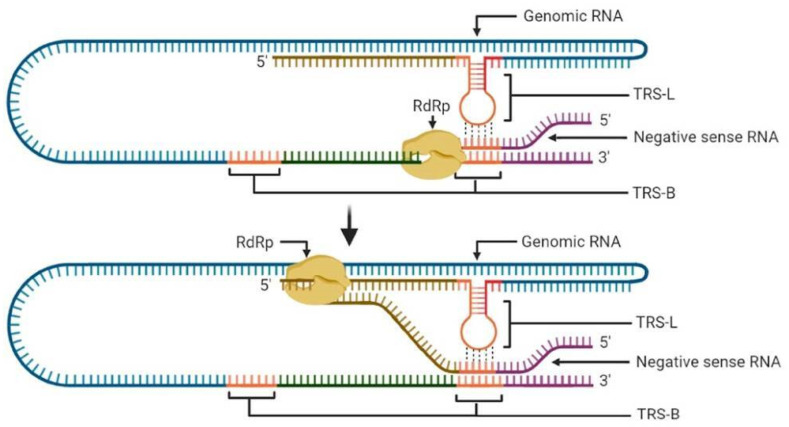
Coronavirus transcription strategy for subgenomic mRNA (sgmRNA) generation. Transcription regulatory sequences (TRSs) are present before each gene, TRS-B, and also before the 5′ leader sequence of the genome (TRS-L). Dotted lines show the base pairing of TRS-B and TRS-L conserved sequences. The base pairing thus leads to a switch in the RNA-dependent RNA polymerase (RdRp) replication strand to the leader sequence present at the 5′ end of the coronavirus genome.

After the initial translation and processing of all the nsp, RdRP along with associated proteins, start to transcribe the viral RNA to produce subgenomic mRNAs (sgmRNAs). SARS-CoV-2 has been shown to produce nine sgmRNAs that encode viral structural proteins. SARS-CoV-2 is also seen to generate a set of nested sgmRNAs of the 3′ end of the genome by a discontinuous mode of transcription similar to other coronaviruses. Complexity is furthered by the transcription of nested RNAs which have been observed to contain a common leader sequence that is only seen at the 5′ end of the viral genome that is at least two ORFs away from the site of sgmRNAs in the genome [22]. The initiation of transcription is regulated by transcription regulatory sequences (TRSs) present before every viral gene and the 3′ end leader sequence of the viral genome. TRSs contain a 6–7 nucleotide long core sequence that is conserved in all TRSs, enabling the negative strand of TRS body (TRS-B) to bind with TRS leader (TRS-L) of the non-transcribed genomic RNA. Factors other than sequence complementarity are understood to be involved in aiding TRS-B and TRS-L binding, such as accessory proteins and double-stranded loop-forming capacity of TRS-L. Upon binding, the RdRp switches to the TRS-L sequence, hence creating a discontinuously transcribed RNA with the leader sequence at the 3′ end of the negative sense, thus producing sgmRNA (Figure 3). These RNAs are then again replicated to create positive-sense strands required for translation [23].

### 3.2. Cellular Pathogenesis

The first interaction of a virus with the host is via its entry receptor. Coronaviruses interact with cell surface proteins using spike protein, a type 1 glycoprotein with two subunits, S1 and S2. S2 acts to facilitate viral envelope fusion, with the cell membrane and the S1 C- and N-terminal domains directly binding to cellular receptors [24]. While the majority of coronaviruses use aminopeptidase N (APN) for initial binding, a subset of viruses, including SARS-CoV and SARS-CoV-2, utilize angiotensin-converting enzyme 2 (ACE-2) as the receptor for cell entry [25,26]. MERS-CoV, on the other hand, binds with dipeptidyl peptidase 4/CD26 to facilitate cell entry [27]. Presently, a few studies have also shown that basigin or CD147 can also act as an alternate entry point for SARS-CoV-2. Co-immunoprecipitation studies have shown that viral spike protein and CD147 bind with each other [28]. The blocking of CD147 by the monoclonal antibody meplazumab has been seen to significantly inhibit viral entry into host cells. A study on 17 patients (NCT number: NCT04275245) conducted in China showed improved clinical outcomes after treatment with meplazumab [29]. The mechanism of CD147 interaction with SARS-CoV-2 is believed to be similar to the ones seen in other viruses such as human immunodeficiency virus (HIV) [30], Kaposi’s sarcoma-associated herpesvirus (KSHV), and hepatitis B virus (HBV) [31], which is through interaction with cyclophilin A (CyPA). Data from hospitalized patients treated with azithromycin showed a reduction in viral load, possibly through the modulation of ligand/CD147 receptor interactions [32]. Azithromycin is known to halt CD147 downstream pathways by inhibiting metalloproteinases. The idea of an entry receptor other than *ACE2* can explain the varied symptoms of COVID-19 but further research is required to validate CD147 and SARS-CoV-2 interaction.

After gaining entry to the host cells, the interplay between host and viral machinery modulates the outcome of the infection. The endoplasmic reticulum (ER) is closely associated with the replication cycle of the coronavirus in several stages, hence it is hypothesized that stress in the ER may occur due to infection by a coronavirus. ER stress induced in coronavirus-infected cells can be due to heavily glycosylated S protein expression [33], thus leading to unfolded protein response (UPR) that ultimately triggers apoptosis by triggering translational inhibition through eukaryotic initiation factor-2α (eIF2α) [34].

Interestingly, positive RNA viruses have been repeatedly shown to synthesize their RNA in virus-induced structures of cellular endomembrane. These organelles are called “replication organelles” [35,36]. Although the exact mechanism of the formation of these vesicles is not understood, there is a consensus on the role of ER membranes in their formation. This has been validated by studies that have observed ER markers such as Sec61α and protein disulfide isomerase (PDI) on the surface of these viral-induced vesicles [37,38,39]. With respect to the pathways involved in vesicle formation, studies have indicated that coatomer protein (COP)-dependent processes of the secretory pathway play a vital role, along with associated factors such as Golgi-specific brefeldin A-resistance guanine nucleotide exchange factor 1 (GBF-1) in the replication of various viral species [40,41]. In SARS-CoV infection, the depletion of COPB1 and GBF1 has been shown to significantly affect viral replication [42,43].

Another set of hypotheses points to the involvement of the autophagy pathway in double-membrane vesicle (DMV) formation, although there are arguments for and against the hypothesis. A study by Denison et al. showed the co-localization of DMVs with microtubule-associated protein light chain 3 and Atg12 proteins that are involved in autophagy [44]. However, another study shows that autophagy-related 5 (Atg5) is not essential [45]. Many proteins have been observed to co-localize with viral genome’s secondary structures, as the 5′ and 3′ ends of the viral genome are involved in viral RNA synthesis regulation. Some of the host proteins involved in viral replication in other coronaviruses studied in this context are summarized in Table 2. These candidates can act as markers for understanding cell and tissue type susceptibility for viral infection. For a recent review on this subject, refer to [46].

The alveolar septa are identified as the primary site of viral infection by histopathological studies. Computerized tomography (CT) scan images reveal the presence of characteristic pulmonary ground-glass opacification even in asymptomatic patients [3,51]. High *ACE2* expression is seen in epithelial cells of alveolar spaces consistent with the SARS-CoV-2-associated destruction of distal airway spaces [52].

## 4. Host–Pathogen Interaction

*ACE2* has been identified as an important host factor receptor mediating binding of the viral spike protein towards the entry of SARS-CoV-2 into the cell [53,54]. Transmembrane protease serine protease 2 (*TMPRSS2*), the cell surface enzyme, cleaves the S protein and primes, thereby regulating the viral uptake [55,56]. Subsequently, the extracellular peptidase domain of *ACE2* recognizes the receptor domain of S protein, providing molecular recognition and infection [55,57]. Followed by endocytosis, translation activation of viral accessory and structural proteins occurs in the cytoplasm. Here, the virus particles are produced and packaged. The fusion of the virion-containing vesicles with the cell membrane releases the virus in the subsequent local environment. This also leads to the activation of various host central cellular pathways and transductional activation of the immune response.

### 4.1. Host Gene and Genetic Network-Driven COVID-19 Modulation

**Figure 4:**
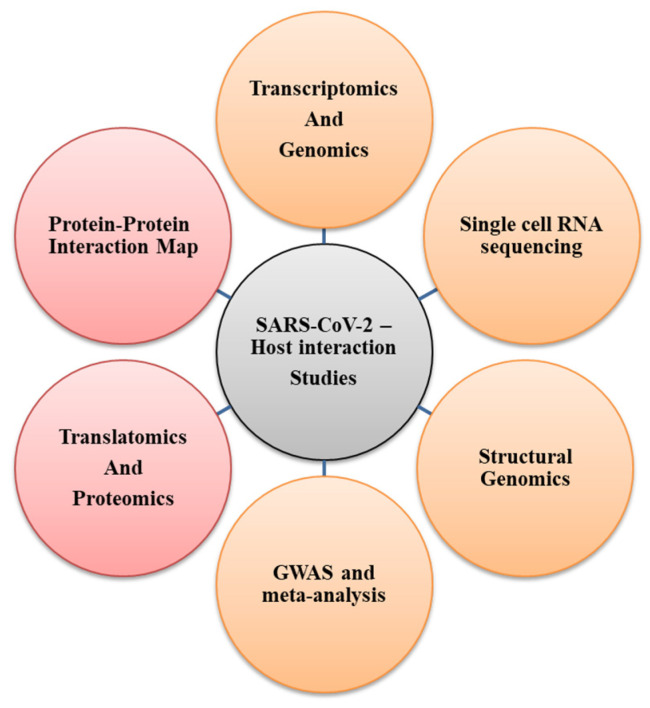
Representation of various experimental techniques used to study SARS-CoV-2 interaction. The interaction with the host by either protein (red) or genomic and transcriptomic (orange) studies.

The integrated systems biology approach is important to support and address the cross-disciplinary understanding towards the elucidation of viral regulation mechanisms using host cellular circuitry, as it contributes to disease severity and mortality. The virus–host interaction has been investigated by several studies to tease out the influence of the virus and host genome, genetic variation, and diversity on infection, pathogenesis, severity, and mortality, among a plethora of other aspects (Figure 4). Translatomics and proteomics of the human cell culture (Caco-2) system infected with a clinical isolate of SARS-CoV-2 revealed the reshaping of the core cellular pathways, including nucleic acid metabolism, translation splicing, and proteostasis. An experimental data-driven approach aided in the identification of viral replication inhibition by ribavirin, NMS-873 [58]. Similarly, the protein interaction map of SARS-CoV-2 and human proteins identified 332 high-confidence protein–protein interactions. The study identified 26 SARS-CoV-2 proteins interacting with human proteins involved in multiple biological processes, including innate immunity, such as NSP13 and ORF9c targeting the interferon and nuclear factor κB (NF-κB) pathways, respectively [59]. Functional regions of SARS-CoV-2 proteins are evolutionarily conserved based on large-scale structural genomics and interactomics roadmaps [60]. Network-based approach analysis of the virus–host interactome revealed high structural similarity among human coronaviruses, although the S-glycoprotein and amino acid sequences were different [61].

**Figure 5:**
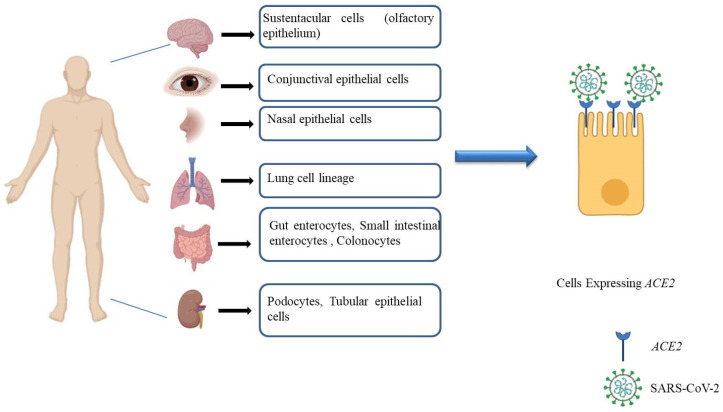
Representative figure of angiotensin converting enzyme 2 (*ACE2)* expression in various cell types of human tissues. It showcases putative and established sites of infection using cell lines, organoid models, and animal models.

A genome-wide association study (GWAS) and meta-analysis of an equal number of participants, approximately 2000 each for positive (COVID 19 and respiratory failure) and controls in cohorts from Italy and Spain, identified genetic susceptibility in the locus 3p21.31 gene cluster and showed ABO blood group system association with respiratory failure [62]. A recent genomic and immunological study suggested the disease severity was mainly attributed to lymphocytopenia and age (host factors) since variation in the viral genome did not confer significant association [63]. Sequence analysis of two Indian isolates of SARS-CoV-2 identified a conversion mutation in the receptor-binding domain (RBD) of the spike protein at position 407 in one isolate. The mutation changed the secondary structure of the protein with the potential alteration of the receptor binding of the virus [64]. Transcriptomic data identified a wide distribution of *ACE2*, and *TMPRSS2* expression across various human tissues in specific cell subtypes, with higher expression levels in various cell subtypes of the lungs, colonocytes, liver cholangiocytes, and nasal goblet secretory cells, among others [65,66]. Expression of *ACE2* and *TMPRSS2* in these cells is indicative of potential sites of infection for SARS-CoV-2, validated experimentally using cell lines and organoid models of cells of different subtypes of tissues, including the brain, colon, gut, ileum, liver, lung, eye, and kidney, and also in animal models, as shown in Figure 5 [67,68,69,70,71,72,73,74,75,76,77]. High expression of *ACE2* observed in intestinal cells [78] is understood to be the reason for COVID-19-associated digestive symptoms such as diarrhea, nausea, pharyngalgia, abdominal pain, and vomiting [79].

### 4.2. DNA and RNA Editing: Impact on Accelerated/Forced Genome Evolution

A gradual process of accumulation of random mutations drives genome evolution. Conversely, active modification of genome-encoded information also accelerates evolution [80,81]. Highly regulated RNA editing plays a crucial role in various cellular pathways, including responses to viral infection and innate immunity.

Host RNA editing has been known to change the base composition and cause viral structural modifications in RNA viruses [82,83,84,85]. The apolipoprotein B mRNA editing enzymes, catalytic polypeptide-like (APOBECs) and adenosine deaminases that act on RNA (ADARs) are two deaminase families expressed in mammalian species, which target single-stranded RNA (ssRNA) and single-stranded DNA (ssDNA) for the deamination of cytidine to uracil (C to U) and the deamination of adenine (A) to inosine (I) on double-stranded RNA, respectively. The rapid increase in various platforms for high-throughput sequencing [86], the sharing of genomic data (GISAID, JHU Dashboard, Nextstrain), and data analytical tools (Viral track; [87]) with open access enable the availability of unmatched resources for the investigation of interactomics of host and pathogen using transcriptome sequencing data.

Analysis of SARS-CoV-2 RNA sequencing datasets of bronchoalveolar lavage fluids from COVID-19 patients confirmed the editing of SARS-CoV-2 RNA by host machinery [88,89]. Although the mutation level percentage observed was low (~1%), it correlated with the rate of RNA editing by the ADAR and APOBEC enzymes in the human genome. This was further validated in the study when the mutational patterns remained consistent in different strains (SARS-CoV and MERS-CoV) of Coronaviridae from human hosts. The observed A to I and C to U nucleotide changes were indicative of the deaminase enzyme-based RNA editing [88].

The RNA editing of the virus would either force it to evolve, as it directly affects the genetic information. ADAR and APOBEC gene family polymorphisms have been studied in depth for their influence in the progression, as well as the susceptibility of microbial (including viral) infection among other human diseases [90,91,92,93,94], thus necessitating the elucidation of role of the identified single nucleotide variants (SNVs) in SARS-CoV-2 genomes for functional study to give us a better understanding of their role in the spread of infection.

### 4.3. Immune Response to SARS-CoV-2

Accumulating evidence suggests that the immune response to SARS-CoV-2 results in innate and adaptive immune cell activation in the infected host. Viral nucleic acids can be recognized as invaders by alveolar epithelial cells and alveolar macrophages by pattern recognition receptor (PRR) families, such as Toll-like receptors (TLRs), retinoic acid-inducible gene-I (RIG-I)-like receptors (RLRs), and nucleotide-binding oligomerization domain (NOD)-like receptors [95,96,97,98]. PRRs detect RNA structure aberrations formed during the replication of the virus, leading to its oligomerization and downstream transcriptional activation of interferon regulatory factors (IRFs) and NF-κB [99]. Two antiviral programs are set in motion due to transcriptional activation of IRFs and NF-κB. Firstly, transcriptional induction of type I and III interferons (IFN-I and IFN-III, respectively) upregulate the IFN-stimulated genes (ISGs) mediating the cellular antiviral defense [100]. Secondly, chemokine secretion orchestrates the recruitment of a subset of leukocytes, including neutrophils [101].

At the site of infection, epithelial cells, endothelial cells, and alveolar macrophages, along with the accumulation of neutrophils [102], release pro-inflammatory chemokines and cytokines. The released interleukin (IL)-1 beta, IL-1ra, IL-2, IL-4, IL-6, IL-7, IL-8, IL-9, IL-10, IL-13, IL-17, monocyte chemoattractant protein (MCP)-1 macrophage inflammatory protein (MIP)-1 alpha, MIP-1 beta, and IP-10 further promote inflammation by recruiting other cells such as monocytes, macrophages, and T cells. The feedback loop for pro-inflammation is established with IFN-gamma produced by CD4+ and CD8+ T cells, resulting in cytokine storm syndrome [103,104,105,106,107,108,109].

Recruitment of these cells in most individuals with mild and moderate cases clears the infection, which lessens the immune response with the recovery of patients. The dysregulation of immune cells in COVID-19 patients [103,104] and characteristic lymphocytopenia in severe cases [105] trigger a cytokine storm [104,105,108,110]. This is supported by the increased levels of IL2, IL7, IL10, IP10, MCP1, MIP1A, and tumor necrosis factor α (TNFα), along with an increased level of granulocyte colony-stimulating factor (G-CSF), fibroblast growth factor (FGF), granulocyte macrophage colony-stimulating factor (GM-CSF), vascular endothelial growth factor (VEGF), MCP-1, platelet-derived growth factor (PDGF), MIP-1 alpha, MIP-1 beta, and IP-10 in comparison to milder/non-intensive care unit (ICU) patients [107].

Although the exact mechanism by which SARS-CoV-2 evades the innate immune response is still unclear, a combination of 5′ cap methylation at the N7 position and sequence mutation (m6A methylation) in the S1 fragment of SARS-CoV-2 may facilitate the evasion of recognition by the *ACE2* binding site [111]. Translational inhibition mediated by non-structural protein 1 (Nsp1) of SARS-CoV-2 effectively blocks the RIG-I-dependent innate immune response [112].

Cytokine storms and increased levels of TNF, among other cytokines, along with local damage, may also cause septic shock. In cases of mortality, respiratory failure is seen to be the leading cause, followed by multiple organ failure, cardiac arrest, hemorrhage, and renal failure [113].

## 5. Role of Host Genetics in Susceptibility to SARS-CoV-2

**Figure 6:**
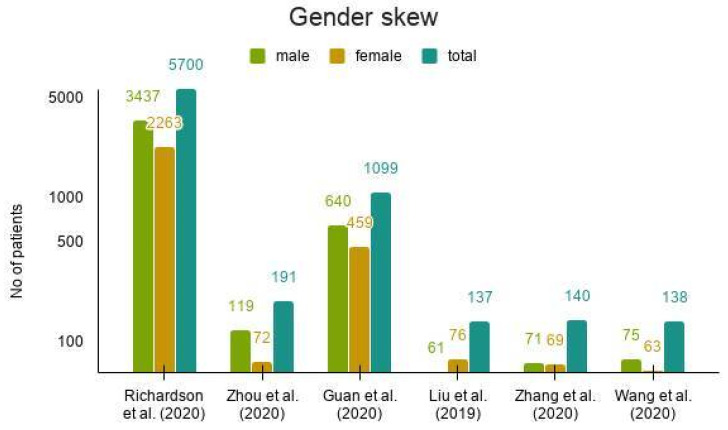
Gender skew among SARS-CoV-2 patients. Male patients are more likely to test positive for SARS-CoV-2 in comparison to female patients [3,102,117,118,119,120].

Disease outcomes of SARS-CoV-2 infection are heterogeneous, depending on several factors. Various hypotheses have been formulated and studied to investigate the cause of observed differences. As is the case with many complex diseases, there is likely a combination of genetic and non-genetic factors responsible for variable clinical symptoms and disease progression in COVID-19. Analysis of epidemiological and demographic data has highlighted the role of age, male gender (Figure 6), history of smoking, and underlying disease comorbidities (cardiovascular disease, hypertension, type 2 diabetes) as factors responsible for disease aggravation [114]. A meta-analysis of 13 studies, including over 3000 patients with COVID-19, corroborated the above observation [115]. However, the occurrence of severe disease or mortality in young adults, previously known to be without any underlying health condition, has presented a glaring discrepancy.

Early studies to explain this variability of symptom severity among people diagnosed with COVID-19 focused on the epidemiology and genomic characterization of SARS-CoV-2 [116]. The role of host genetics in impacting the severity and susceptibility to COVID-19 received less attention in the initial phase. However, more recent studies emphasizethe importance of elucidating the role of host genetic variants with variability in clinical outcome and viral genome features. Researchers in Shanghai, who examined clinical, molecular, and immunological data from over 300 COVID-19 patients, highlighted the evolution of the ancestral SARS-CoV-2 virus into two major lineages with differential exposure history. However, viral genetic variations did not exhibit significant differences with respect to virulence and clinical outcomes. The study attributed the variation of disease severity and disease outcome to host factors rather than viral genetic variation [63].

### 5.1. Host Genetic Markers Associated with COVID-19 Susceptibility

Large variability in the clinical features of COVID-19 point towards the selectivity of SARS-CoV-2 across human genetic variations [121]. The underpinnings of susceptibility to infection may be partially explained by variations in host genes associated with viral replication processes and immune modulatory processes. Table 3 summarizes some of the prominent host genetic loci associated with susceptibility and disease outcomes for SARS-CoV and SARS-CoV-2.

The focus on viral dynamics in the host helped to establish the *ACE2* gene as the human receptor for the novel coronavirus, much like the earlier SARS-CoV [14,122,123]. Differential expression patterns of the *ACE2* receptor in different ethnicities and its variation with age and gender are believed to affect the susceptibility, symptoms, and outcome of COVID-19 [124,125]. A study by Chen et al. further established a negative correlation between *ACE2* expression and COVID-19 fatality at both population and molecular levels [126]. The individual variation in *ACE2* levels is also determined by inducing factors such as smoking. Leung et al. found that *ACE2* expression is increased in the small airway epithelia of smokers, predisposing them to a higher risk of infection with SARS-CoV-2 [127,128]. Variation in disease outcome, including susceptibility to fatal lung injury and myocardial injury, is also believed to be linked to distributional and functional aspects of the *ACE2* receptor in populations [129].

Much like other viral infections, COVID-19 pathogenesis is also a result of the interplay between viral transmission dynamics and host response. Studies done in the past have established the role of human leukocyte antigen (HLA) alleles in conferring differential susceptibility [130] and severity [131] for several viral infections. A comprehensive in silico analysis attempted to construct an HLA susceptibility map for SARS-CoV-2 [132]. The study included patients of Asian descent and found that the presence of the HLA-B*46:01 allele was associated with severe cases of infection. However, the study lacked validation with respect to clinical data and was entirely based on computational analysis.

Susceptibility to viral infections has been previously linked to antigenic determinants of ABO blood groups in the case of SARS-CoV. Individuals with blood group O were less likely to be infected by the virus than non-O blood groups (odds ratio, OR, of 0.18) [133]. Extrapolating this knowledge, Zhao et al. investigated the relationship between ABO blood groups and the incidence of COVID-19. The preprint article reported that blood group A had a significantly higher risk for COVID-19 (OR 1.21; *p* = 0.027) and blood group O had a significantly lower risk for SARS-CoV-2 infection (OR 0.67; *p* < 0.001) when compared to other blood group types. They also found a higher mortality risk associated with blood group A in comparison to blood group O (OR 1.482; *p* = 0.008), while blood group O was found to carry a lower mortality risk (OR 0.660; *p* = 0.014) when compared to non-O blood groups [134]. The findings were replicated separately in another study by Li et al. [135].

**Table 3:** Summary of human genetic loci related to SARS-CoV-2 infection and disease outcomes.

Functional Group	Associated Gene/Loci	Key Findings	References
Host target proteins	*ACE2* (cell entry receptor for SARS-CoV-2)	*ACE2* localization on X-chr, SARS-CoV-2 infection in males. *ACE* D allele (In/Delpolym), hypoxemia in SARS-CoV-1 patients.	[136,137]
	*TMPRSS2* (cell surface serine protease involved in S protein priming)	rs12329760, genetic susceptibility to SARS-CoV-2.	[136]
Host immune response genes	*HLA* (human leukocyte antigen genes)	HLA-B* 4601, severity of SARS-CoV-1 & SARS-CoV-2 infection	[132,138]
	*IL-6* (interleukin-6, a pro-inflammatory mediator)	*IL-6-174C*, higher IL-6 level, pneumonia severity (C allele vs. G allele).	[139]
Virus targeting host proteins	*OAS1*, viral RNA degradation and inhibition of viral replication	rs2660 and rs3741981, susceptibility to SARS-CoV-1.	[140,141]
	*MBL*, innate immunity, as an ante-antibody before Ig response	rs1800450 (*MBL*) along with *CCL2* rs1024611, associated with SARS-CoV-1 susceptibility. Serum MBL was lower in SARS-CoV-1 patients than controls.	[142,143,144]
	FCGR2A, encodes Ig receptor present on surface of phagocytic cells	Homozygosity for *FCGR2A* rs1801274 and *FCGR2A*-RR131 predispose towards severe SARS-CoV-1 infection.	[145]
	MX1, antiviral protein induced by IFN alpha and beta	*MX1* rs2071430, a polymorphism in the *MX1* gene promoter associated with lower risk of SARS-CoV-1 infection and hypoxemia.	[140,142,146,147]

### 5.2. International Efforts in Studying Host Contribution to SARS-CoV-2 Susceptibility

COVID-19, as a disease with global ramifications, has underscored the urgency and need for a global effort and transparent data sharing across countries to enable better understanding and the development of containment strategies for the virus. At the international level, the focus of COVID-19 research has drifted towards finding the host genetic determinants responsible for the large variability in disease outcome and confer susceptibility or resistance to the development of critical illness and or death. These investigations build upon the resources of already existing public and private databases, while also establishing new study cohorts. Recognition of this need has driven the launch of many collaborative and multi-country efforts to bring together the genetic community across the world to generate, share and analyze data to discover the host genetic contributors of COVID-19 susceptibility, severity, and disease outcomes (Table 4).

## 6. COVID-19 and CoMorbidities

**Figure 7:**
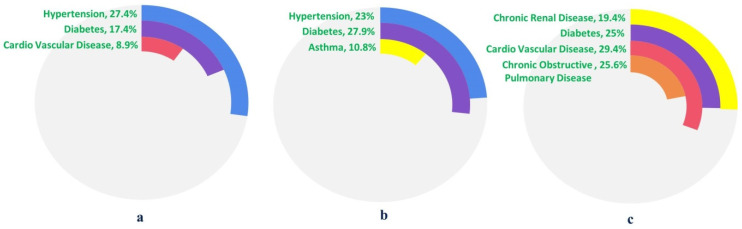
Comorbidity predisposition across (**a**) SARS-CoV-2, (**b**) MERS, (**c**) H1NI, all subtypes. Differential percentage of comorbidities associated with viral infection types indicate their potential functional role in disease outcome.

The global pandemic due to SARS-CoV-2 has once again challenged the health care system worldwide and multi-dimensional coverage of this on-going crisis revealed the significance of healthy host response in combating infectious diseases. Host competency is often considered a key factor in deciding the prognosis of a disease. Hence, disease trajectories and resolution are not only governed by pathogen virulence but also attributed to the overall health of the host. Comorbid conditions antagonize the host response against the index disease that furthers the exaggeration of otherwise treatable symptoms (Figure 7).

Earlier epidemiological correlative studies with other RNA viruses like influenza and dengue emphasize the strong correlation between the existence of comorbidity or polymorbidity with disease severity [148,149]. A comprehensive analysis of cumulative cases of influenza subtype strains in 12 Catalan hospitals during 2010–2016 establishes a strong correlation. In influenza subtype B infection, ICU admission was significantly associated with chronic obstructive pulmonary disease (COPD), whereas those with chronic cardiovascular diseases were the least likely to be admitted to an ICU [148]. Small sample sizes have been an impeding factor for any logical deduction in the case of MERS, however, in a study of 226 patients, diabetes followed by hypertension were frequent comorbidities [150]. To date, SARS-CoV-2 has spread in more than 200 countries and has been able to cause systemic infection in human hosts, and the most common complications are ARDS, acute renal injury, acute coronary injury, etc. An exhaustive analysis which includes 27 different studies with a total of around 22,000 cases has shown some interesting facts underlying an important aspect of SARS-CoV-2 pathophysiology [151]. The prevalence of comorbidity worsens the symptoms 10-fold but does not always translate into a fatality. Fifty-seven percent of patients have been reported to possess some comorbid condition, with hypertension and diabetes among the most prevalent ones [151].

### 6.1. Diabetes

The syndromic nature of diabetes is manifested through its effects on many organs [152]. In previous outbreaks, diabetes is known to confer an increased risk of infection, and patients having pre-existing conditions of diabetes were reported to have more severe outcomes than patients with no morbidities [153,154]. Diabetic patients are most susceptible to dermatological and urinary tract-associated complications, but its systemic nature has exposed much vulnerability in various viral outbreaks [155,156,157,158]. Diabetes is accompanied by increased levels of several pro-inflammatory cytokines, including TNF-α, IL-6, and IL-1β [159]. An enhanced flux of electrons to the respiratory chain causes electrons to leak and form reactive oxygen species and glycation adduct, which is capable of eliciting the pro-inflammatory response through nuclear localization of NF-κB [160,161,162]. During hyperglycemia, certain glycolytic intermediates like dihydroxyacetone phosphate accumulate and eventually transform into diacylglycerol. Protein kinase C is activated through diacylglycerol and results in the synthesis of TNF-α and nitric acid synthase, exaggerating an already activated pro-inflammatory response that can lead to altered tissue pathophysiology [163,164]. At the cellular level, mobility and phagocytosis were found to be hampered in polymorphonuclear leukocyte (PMN) cells isolated from diabetic patients [165,166]. Hyperglycemia is also a cause of a reduction in levels of adhesion molecules like ICAM-1 and VCAM-1 that systemically affect cellular movements to counter various viral and bacterial attacks [166].

### 6.2. Hypertension

The interplay of diverse genetic factors and environmental conditions determines the complex trait of blood pressure [167]. Abnormal variations in blood pressure are a cause that predisposes many tissues to further damage by internal and external insults. These changes in hypo- or hypertensive patients subject them to severe clinical complications that are likely a cause of a strong association of hypertension with SARS-CoV-2 infection, which is to some extent due to the higher expression profile of ACE2, a surface receptor necessary for SARS-CoV-2 entry [122,168,169]. In other respiratory disorders which cause a collection of symptoms, together called ARDS, hypertension is already a well-established comorbidity and can be fatal. The interplay between hypertension and COVID-19 could lead to a synergistically increased risk for adverse outcomes due to a decrease in levels of expression of ACE2, due to the use of its of inhibitors that facilitate further entry of SARS-CoV-2 [170]. Understanding the underlying pathophysiology in hypertension is aided by the development of genetically developed and experimentally induced animal models (spontaneously hypersensitive rat, Dahl salt-sensitive rat), characterized by excessive sympathetic activation, glomerular injuries, and cardiac hypertrophy. Pathophysiology in the above-mentioned animal models emphasized the infiltration of immune cells, presence of oxidative stress, and enhanced levels of pro-inflammatory cytokines, which may adversely affect SARS-CoV-2 infection outcomes, similar to diabetes [171,172,173,174,175].

## 7. Co-Infection Associated with SARS-CoV-2

**Figure 8:**
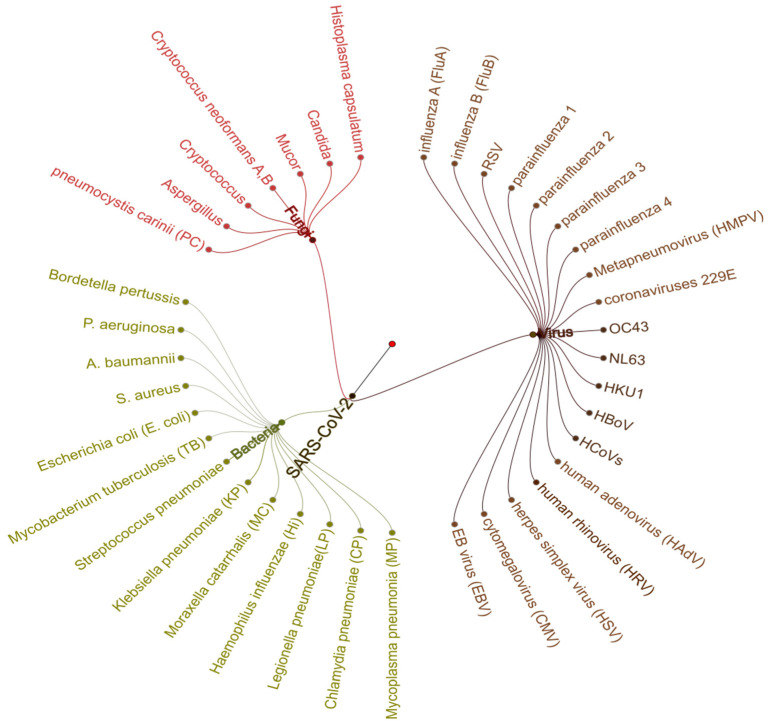
SARS-CoV-2-associated co-pathogens reported in various studies. Accounting for all the viruses, bacteria, and fungal pathogens, we found a total of 40 respiratory co-pathogens reported by various studies [117,182,184,185,186,187].

**Figure 9:**
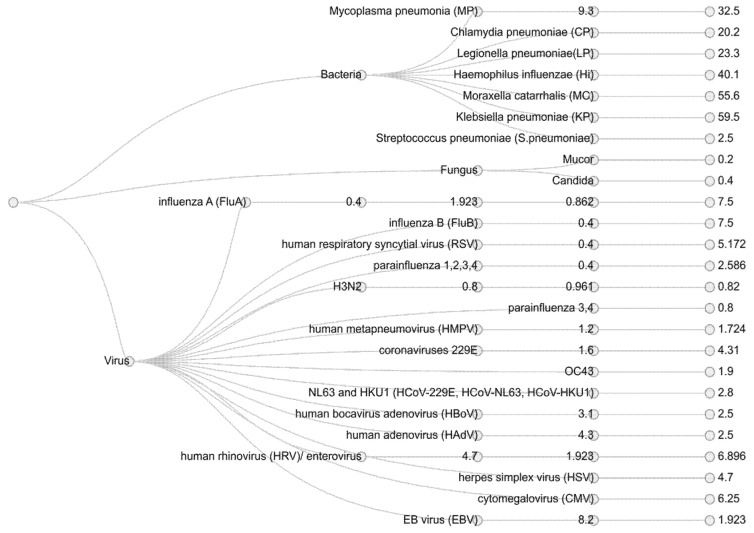
A co-pathogen dendrogram representing the classification of reported co-pathogens associated with SARS-CoV-2 infections. The data for this representation include four different studies [183,184,191,192], with 104, 116, and 40 samples, respectively.

Viral, bacterial, and fungal co-infections in hospitalized SARS-CoV-2 patients are poorly understood to date. It is essential to differentiate between hospital-acquired infection and the co-presence of other pathogens. This can be either a secondary infection due to a compromised immune system or pre-existing commensal/s with probable functional roles. Thus, samples collected during the first report to the hospital would be ideal to study the role of co-infections. Co-infection can stimulate and inhibit the immune system of the host, altering the disease profile upon co-infection with other microorganisms [176,177,178,179]. It has been observed during previous influenza pandemics that the bacterial and viral co-infections were one of the major causes of mortality. For example, in community-acquired pneumonia (CAP) cases, the influenza-related bacterial infection alone may account for up to 30% of total cases [180]. Several reports indicate that viral infections have some bearing on infections/diseases and increase the susceptibility of patients towards other diseases [181]. As the viral respiratory infections are mostly of short duration, so the majority of the studies have focused on chronic infections such as HIV and hepatitis A and B; thus presenting an opportunity to explore and elucidate the role of co-pathogens during SARS-CoV-2-induced ARDS [182,183]. The prevalence of co-infection is not uniform among SARS-CoV-2 patients across studies. However, it is observed that around 50% of the non-survivors were carrying the co-pathogens [183]. In the majority of the studies, the co-pathogens of SARS-CoV-2 included mainly bacteria and viruses (Figure 8 and Figure 9).

Clinically, it is challenging to differentiate between SARS-CoV-2-related viral infection and other possible bacterial and fungal infections. Several studies from Wuhan have reported secondary bacterial infections in SARS-CoV-2 patients [117,182,186,187]. Studies around co-infection will help to determine the various viruses in the etiologic treatment of respiratory diseases and can potentially suggest various therapeutic aspects of it.

Among all the other influenza pandemics, SARS-CoV-2-positive samples show co-infections for *Aspergillus* spp., *Staphylococcus aureus*, *Streptococcus pneumoniae*, and *Haemophilus influenzae*. However, the source of infection remains unclear to date. Influenza A virus was one of the common co-pathogens observed in SARS-CoV-2 patients. It is also reported that patients with influenza A virus showed false-negative results for SARS-CoV-2 infections [188,189]. Other than influenza, rhinovirus/enterovirus has also been reported by several studies. Similarly, *Legionella pneumophila*, *Mycoplasma pneumoniae*, *S*. *pneumoniae*, and *Chlamydia pneumoniae* were identified as bacterial co-pathogens but very few studies have found a statistical significance for the presence of these co-pathogens [190].

A retrospective study by Xiaojuan Zhua et al. highlighted that there are different co-pathogens present in patients with symptomatic, mild, moderate, and severe cases of COVID-19, with a predominance of bacterial pathogens. The reported co-pathogens *Escherichia coli*, *Klebsiella pneumoniae*, *H*. *influenzae*, *S*. *pneumoniae*, *Aspergillus* spp., Epstein–Barr virus (EBV), and *S*. *aureus* were common for all four categories, but a higher incidence of co-infections was reported in severe cases of COVID-19 [188].

## 8. CRISPR-Based Interventions for COVID-19

**Figure 10:**
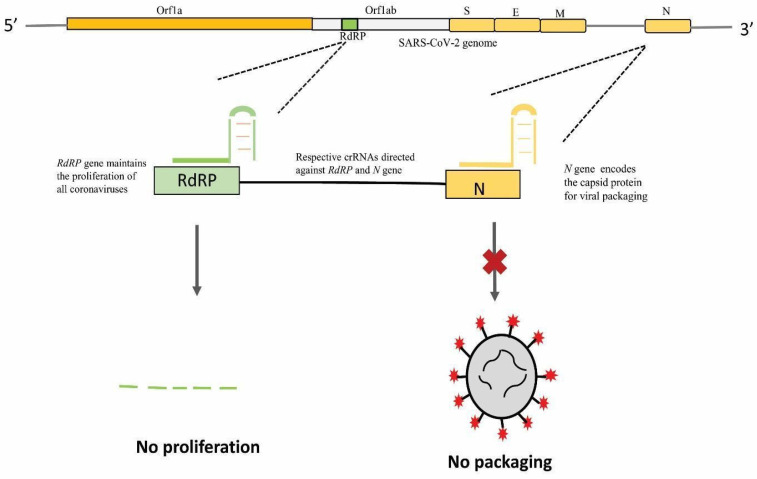
Clustered regularly interspaced short palindromic repeats (CRISPR) based antiviral targeting strategy, PAC-MAN. The crRNAs directed against the RdRP gene and *N*-gene of SARS-CoV-2. Due to the inhibition of the RdRP gene, no proliferation of the viral RNA genome will occur and due to inhibition of the *N*-gene, no viral genome packaging occurs.

**Figure 11:**
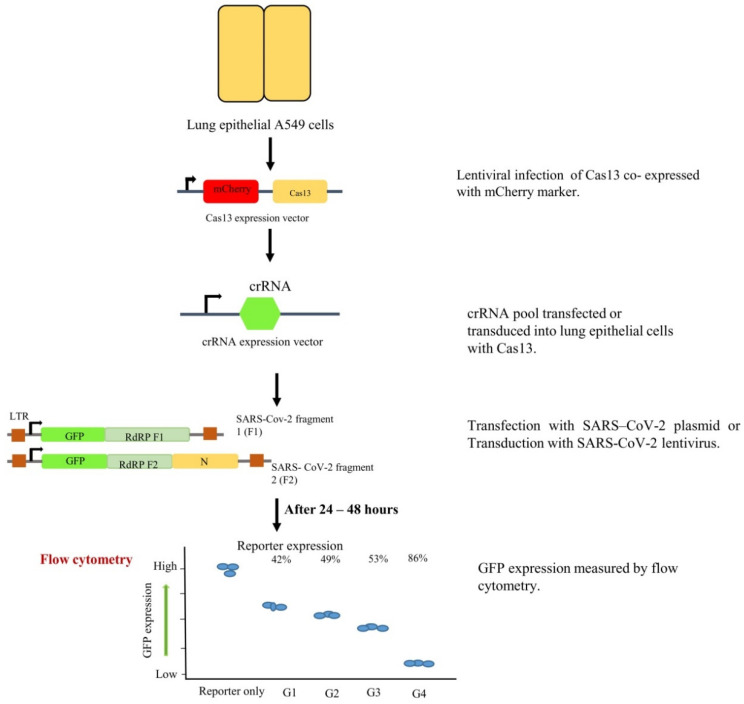
Schematic representation of experimental workflow for crRNA-based inhibition of SARS-CoV-2. A stable A549 cell line expressing Cas13d through lentiviral infection was developed. Transduction with the crRNA pool was done, followed by transfection or transduction of Cas13d A549 cells with SARS-CoV-2 reporters. After 24 hours of reporter transfection, flow cytometry was performed to assess GFP protein expression.

The clustered regularly interspaced short palindromic repeats (CRISPR) system has been extensively used since its discovery and has genome-wide applications [193]. A recent study at Stanford University, by Stanley Qi et al., devised a strategy based on CRISPR-Cas13 called prophylactic antiviral CRISPR in human lung epithelial A549 cells (PAC-MAN) for the inhibition of SARS-CoV-2. The Qi lab designed a group of CRISPR RNAs (crRNAs) that targeted conserved regions in the viral genome and spotted functional crRNAs directed against synthetic SARS-CoV-2 (Figure 10). It revealed that the crRNA pool targeting RdRP was able to suppress reporter expression to some extent as compared to control. The study found that Cas13d PAC-MAN could be used as a therapeutic tool to target and degrade SARS-CoV-2 sequences in human cells by designing crRNAs for the efficient inhibition of SARS-CoV-2 (Figure 11). However, the possible roadblock for using the PAC-MAN strategy is in vivo delivery of the CRISPR-Cas system to cells and the use of live SARS-CoV-2 instead of the synthetic virus [194].

The CRISPR/Cas9-mediated gene editing approach may be used for engineering B cells, as proposed by Faiq et al. He further proposed that the substitution of endogenously encoded antibodies in human B cells with antibodies directed against SARS-CoV-2-specific antigens generated via the CRISPR/Cas9 system may prove to be an efficient strategy. This might be a promising tool to develop long-lasting and effective vaccines against SARS-CoV-2 and other pathogens, including HIV, EBV, ebola, and dengue [195].

### CRISPR-Based Detection Kits

**Figure 12:**
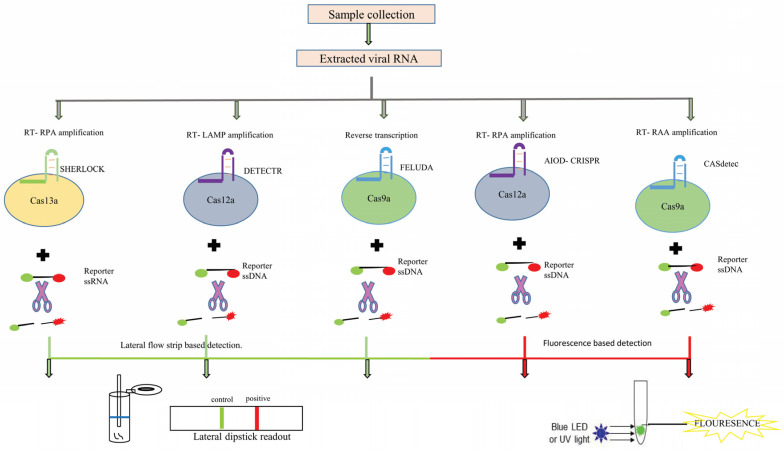
Workflow comparison of different CRISPR-based detection assays. Different Cas variants are used for their efficiency of detection with time as an important variable.

Researchers across the globe are racing against time to develop rapid and innovative COVID-19 detection kits. Besides its use as an antiviral strategy, CRISPR-based systems are being harnessed as a diagnostic tool to detect SARS-CoV-2 infection. A detection tool based on CRISPR employs an isothermal amplification procedure within it, such as loop mediated isothermal amplification (LAMP), recombinase polymerase amplification (RPA), or others (Figure 12). CRISPR-based detection kits utilize the CRISPR/Cas system combined with a reporter molecule to detect the presence of SARS-CoV-2. The readout signal is usually measured by detecting fluorescence or colorimetric change on lateral flow strips (dipsticks). Some of the most promising CRISPR-based detection kits in use are shown in Table 5.

## 9. Pharmacological Interventions in SARS-CoV-2

### 9.1. Drugs and Therapeutics

Since the outbreak of COVID-19, considerable research effort has gone into unraveling the unique viral structure, its lifecycle, and its pathogenesis to decipher key targets that may be crucial in inhibiting the spread of SARS-CoV-2. Scientists across the globe have been struggling to develop and design efficacious treatments to contain the disease. Most of the effort in developing a treatment for SARS-CoV-2 is driven by relying on the experience and knowledge gained previously with SARS and MERS viruses, all of which belong to the family of *Betacoronavirus* [185].

Due to the absence of definitive treatment for SARS-CoV-2 to date, clinical management of the disease is primarily aimed at symptomatic relief and supportive care [202]. The research fraternity, including academic labs and the pharmaceutical industry, are working hard to find a cure that may contain the spread of SARS-CoV-2 in humans. The development of novel drugs/therapeutic agents is an arduously long process, taking years, from preclinical to the clinical trial stage, including the determination of its safety and efficacy. Given the urgency of the current situation, most of the global effort is directed towards the identification and evaluation of known drugs and therapeutics that may be repurposed for the treatment of COVID-19. An added benefit of repurposed drugs is the ease of accessibility, known pharmacokinetic and pharmacodynamic parameters, well-established safety profiles, and dosing regimens [203].

**Figure 13:**
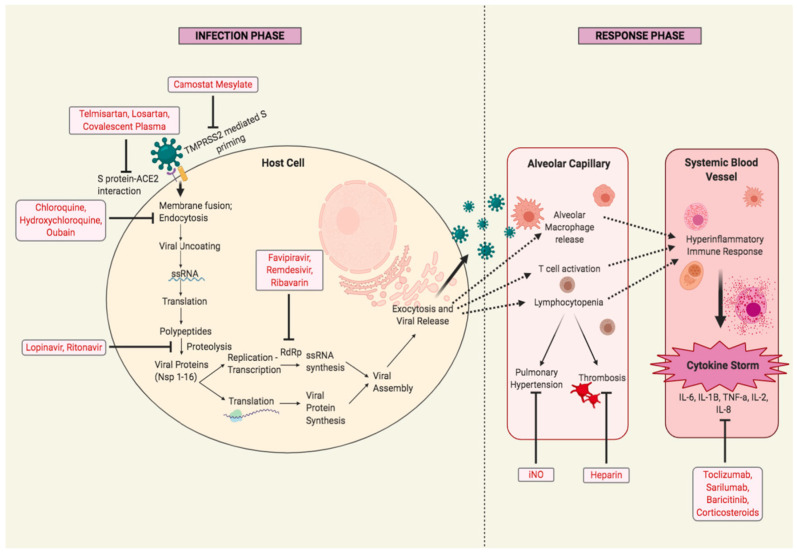
Schematic representation of the SARS-CoV-2 infection cycle within host cells and virus-induced host immune response and possible targets of anti-COVID-19 drugs. The left section represents the infection stage wherein, SARS-CoV-2 infects the host cells. Telmisartan and losartan are *ACE2* blockers that inhibit viral binding to it. Convalescent plasma has antibodies against the S protein of SARS-CoV-2, dampening S protein–ACE2 interaction. Camostat mesylate inhibits *TMPRSS2* and prevents S protein priming for binding to ACE2. Chloroquine, hydroxychloroquine, and ouabain inhibit viral fusion with host cell membranes and endocytosis. Lopinavir/ritonavir is an inhibitor of 3CL protease, blocking proteolysis of viral polypeptide chains. Favipiravir, remdesivir, and ribavirin are inhibitors of RNA-dependent RNA polymerase (RdRp) which is required for the replication of the viral genome. The right section depicts the host immunological response to SARS-CoV-2 infection at the pulmonary and systemic level. Here, iNO reduces pulmonary hypertension by inducing vasodilation. Heparin is an anticoagulant that blocks thrombus formation. Toclizumab and sarilumab are monoclonal antibodies that prevent IL-6-mediated inflammation. Baricitinib inhibits the JAK/STAT signaling pathway involved in the inflammatory response. Corticosteroids also work to reduce inflammation. Abbreviations: S protein: spike protein; ACE2: angiotensin converting enzyme 2; *TMPRSS2*: type 2 transmembrane serine protease; ssRNA: single-stranded RNA; Nsp: non-structural proteins; RdRp: RNA-dependent RNA polymerase; iNO: inhaled nitric oxide; IL: interleukin; TNF: tumor necrosis factor.

The most promising therapeutic options currently being explored against SARS-CoV-2 include agents targeting critical steps in the viral life cycle, such as viral entry into host cells involving membrane fusion and endocytosis, key structural proteins, and enzymes involved in processes such as viral replication, transcription, assembly, and release of the virus [204,205]. Another major line of treatment includes adjunctive therapies that target the fundamental immune regulation pathways affected in response to SARS-CoV-2 infection. Figure 13 represents the target points of various drugs and adjunctive therapies of some of the most promising treatments that are in an advanced stage of clinical trials.

### 9.2. Vaccines

**Figure 14:**
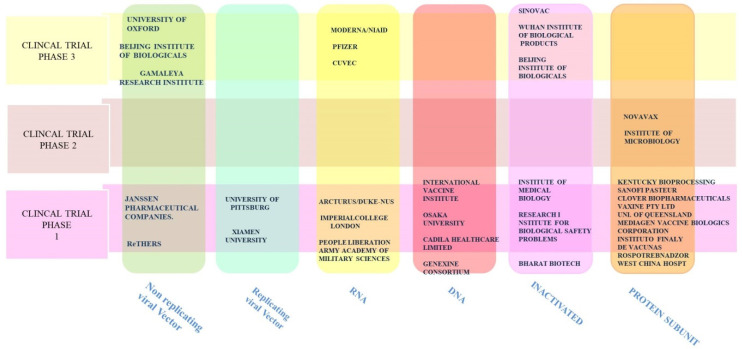
At the moment, 35 candidate vaccines are currently in various phases of clinical trials. Vertical bars represent a template and at least one of the academic institutions or industries involved in the process of developing a vaccine. Horizontal bars represent clinical trial phases. Source: DRAFT landscape of COVID-19 candidate vaccine, WHO.

Vaccines, conventionally and traditionally, are considered major ammunition in countering viral infections and providing protection against them. Previous work on SARS-CoV and MERS-CoV has provided crucial scientific evidence in support of the potent immunogenicity of S protein in raising neutralizing antibodies when compared with other proteins like M protein, N protein, and E protein [206,207,208]. In both MERS and SARS-CoV, full-length S1 protein was found to be capable of eliciting the adequate immune response but brought along some adverse physiological reactions such as focal necrosis in the liver and pulmonary perivascular hemorrhage in animal models, leading to the evolution of more focused programs revolving around subunit vaccines with the RBD as a potential immunogen [208,209,210,211]. Nevertheless, all possible platforms (replicating and non-replicating viral vector vaccines, DNA and RNA vaccines, inactivated and protein subunit vaccines) are being currently explored and are in various phases of clinical trials (Figure 14). Herein, we discuss preclinical studies of three vaccines that have evolved using different templates for vaccine development. In phase 3 of clinical trials, the adenovirus-based ChAdOx1nCoV-19 vaccine encodes full-length S protein and is being developed by the University of Oxford. In rhesus macaques, the ChAdOx1nCoV-19 vaccine reduces the viral load in bronchoalveolar lavage fluid and the lower respiratory tract and also prevents pneumonia. A balanced TH1/TH2 humoral and T cell response was observed with a higher TH1-type cytokine titer after the first injection [212]. Using the RNA platform, the National Institute of Health partnered with Moderna Inc. to produce an mRNA-based vaccine expressing the prefusion-stabilized SARS-CoV-2 S protein trimer (rnRNA-1273). In preclinical studies in mice, mRNA-1273 was found to be potent enough to elicit significant levels of neutralizing antibodies without any sign of vaccine-associated enhanced respiratory disease [213]. PiCoVacc is another candidate in advanced stages of clinical trials, using an inactivated virus developed by the China-based company Sinovac. PiCoVacc, in a combination of alum adjuvant, was mixed and injected into mice to assess the immune correlates. Both S protein and RBD domain-specific antibodies were observed with significant levels in sera [214].

## 10. Future Perspectives

**Figure 15:**
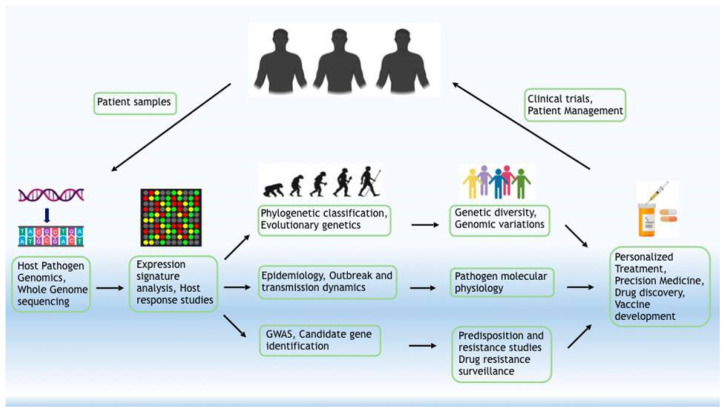
Application of genomics in the management of infectious diseases. The approach integrates the idea of “discover–understand–mechanistic elucidation–application” towards the bigger goal of public health with patient management at the core.

Targeted and large-scale genomic studies provide valuable insights into the gene, pathway, and expression shifts, which may render people differentially susceptible to infection from SARS-CoV-2. When combined with gene expression datasets and drug response studies, the study of host genomic sequences further encourages the search for candidate compounds to target the viruses as they infect human cells. Knowledge of virus dynamics combined with host response is essential for formulating strategies for antiviral treatment, vaccinations, and epidemiological control of COVID-19 (Figure 15).

The Indian population is a genetically heterogeneous group comprising more than 1.4 billion people in over 4000 communities, several endogamous groups, and natives of many ethnicities. The Council of Scientific and Industrial Research (CSIR) led the first large-scale comprehensive study of the of the Indian population’s genomic substructure in the form of the Indian Genome Variation Consortium. The study aimed at cataloging the variations in over a thousand genes implicated in disease and drug response, in 15,000 individuals drawn from Indian subpopulations for the discovery of predictive markers to address the questions about ethnic diversity of the Indian population [215,216]. The IGVBrowser containing a vast database of Indian subpopulations may serve as a useful resource for driving studies on host genetics [216]. The basal level variation data may also explain the observed differences in mortality and morbidity rates across Indian states, mostly characterized by the presence of distinct ethnic groups.

A program on host genetics building on the information from the IGVBrowser database for the Indian population (similar for other population-level databases) will be a huge step towards addressing the spread of the disease and minimizing the costs of diagnosis and treatment. An insight into potential host contributors to SARS-CoV-2 susceptibility and characterization of genetic markers for COVID-19 is essential for many reasons. Firstly, it will allow the development of a prognostic stratification tool to determine patients most at risk of developing severe disease and efficient patient management. Secondly, following the development of a vaccine against SARS-CoV-2, individuals at high risk could be prioritized for vaccination. Last but not least, it will also give a boost to treatment personalization, precision medicine, and drug discovery.

A pan-India genomic study for COVID-19 would serve as a primer for other infectious disease outbreaks in the future. This will also inch the Indian scientific community one step closer towards setting up a national biobank for collecting tissue specimens from patients, along the lines of those existing in the USA and European nations. A biobank for the genomic profiling of patients containing data for thousands of participants will provide empirical evidence to improve clinical care during this pandemic and later.

## 11. Conclusions

SARS-CoV-2 is not the first virus (pathogen) to pose a health scare for the public but, possibly the footprint of SARS-CoV-2 infection has persuaded us to work towards an integrative approach. Specifically, the frequency with which the RNA viruses have led to health challenges requires broad understanding of the infection mediated by RNA viruses as well as the virus themselves. Learnings from the current situation would potentially feed into long-term understanding of the host–pathogen paradigm.

## Figures and Tables

**Table 1:** Nextstrain clade information.

Clade	Primary Countries	Mutations	Max Frequency (2020)
19A	Asia: China/Thailand	Root clade	65–47% globally in January
19B	Asia: China	C8782T T28144C	28–33% globally in January
20A	N America/Europe/Asia: USA, Belgium, India	C14408T A23403G	41–46% globally April-May
20B	Europe: UK, Belgium, Sweden	G28881A G28882A G28883C	19–20% globally Mar-April
20C	N America: USA	C1059T G25563T	19–21% globally April

**Table 2:** Host proteins interacting with viral replication.

Protein	Coronavirus	Proposed Interaction	Protein Function	References
Annexin A2	Infectious bronchitis virus (IBV)	Regulates frameshifting efficacy	RNA binding	[47]
MADP1	SARS-CoV, IBV	Binding to viral 5′UTR	RNA splicing	[48]
NONO	IBV	Interacts with the nucleocapsid protein	RNA splicing	[49]
hnRNP A1	Mouse hepatitis virus (MHV)	Binding to intergenic ORF7 regulatory region and negative-strand leader	RNA splicing, transport	[50]

**Table 4:** Summary of global efforts to study the role of host determinants in SARS-CoV-2 infection and pathology.

Study Name and Location	Objectives	Proposed Methodology	Study Population	Web Link
**COVID-19 Host Genetics Initiative** (global collaborative effort involving over 200 studies across continents).	Platform to generate, share, and analyze data to identify the genetic determinants of COVID-19 susceptibility, severity, and outcomes.	Common and rare variants identified through GWAS array, exome and genome sequencing will be considered for bioinformatic and statistical analysis. Many studies may also include immune and antibody profiling.	Includes both retrospective and prospective cohort studies. The retrospective collection group includes the retrieval of genetic data from existing biobanks and health care systems. The prospective collection studies actively enroll incoming COVID-19 patients.	https://www.covid19hg.org/
COVID Human Genetic Effort (multi-country effort led by Rockefeller University)	Identify the monogenic inborn errors of immunity, which predispose previously healthy individuals younger than 50 years old and even older individuals without any overt comorbidities to life-threatening COVID-19. To identify monogenic variations that make individuals resistant SARS-CoV-2 infection.	Whole exome sequencing (WES) and whole genome sequencing (WGS) to identify candidate disease-causing variants. Functional biochemical studies to characterize the role of candidate variant genes and their effect on cellular immunology.	Previously healthy young patients (< 50 years old) with severe COVID-19 and their family members, and seropositive individuals who have remained asymptomatic as controls. Individuals not infected by SARS-CoV-2 despite repeated exposure, as confirmed by lack of disease and antibody titers.	https://www.covidhge.com/about
GenOMICC COVID-19 study (GenOMICC Consortium led by the University of Edinburgh)	To find out whether people’s genetics affect their experience of COVID-19.	Whole genome sequencing of DNA from study subjects.	20,000 SARS-CoV-2-infected patients with severe symptoms of COVID-19; 15,000 SARS-CoV-2 infected individuals who are asymptomatic or display mild symptoms as controls.	https://www.genomicsengland.co.uk/covid-19/
IMPACC study (at 10 research sites across the United States)	To determine how certain immunological measures correspond to, or may even predict, the clinical severity of COVID-19. To identify new targets and optimal timing for experimental treatments.	Immunological and serological analysis, GWAS.	2000 individuals hospitalized with COVID-19 will enroll in the study within 36 h of admission and will be followed up to one year.	https://www.niaid.nih.gov/clinical-trials/immunophenotyping-assessment-covid-19-cohort-impacc

**Table 5:** CRISPR-based detection assays.

Test Name	Cas Involved/Target	Sample Type	Time	Features	References
Specific high-sensitivity enzymatic reporter unlocking (SHERLOCK)	Cas13a/*S*-gene and *Orf1ab*-gene	Nasopharyngeal swab	60 min	Cleaves ssRNA reporter. Lateral flow strip-based detection.	[196]
DNA endonuclease-targeted CRISPR trans reporter (DETECTR)	Cas12a/*E*-gene and *N*-gene	Nasopharyngeal/Oropharyngeal swab	30 min	Cleaves ssDNA. Lateral flow strip-based detection.	[197]
Combinatorial arrayed reactions for multiplexed evaluation of nucleic acids (CARMEN)	Cas13a	Plasma, nasal, or throat swabs	<8 h	Detects ssRNA. Fluorescent readout.	[198]
All-in-one dual CRISPR-Cas12a (AIOD-CRISPR)	Cas12a/*N*-gene	Nasopharyngeal swab	40 min	Cleaves ssDNA. LED/UV-based detection.	[199]
(FnCas9 editor linked uniform detection assay) (FELUDA)	FnCas9/*NSP8* and *N*-gene	Nasopharyngeal swab	90 min	Cleaves ssDNA. Lateral flow strip-based detection.	[200]
CRISPR-Cas12b-mediated DNA detection (CASdetec)	Cas12b/*RdRP* gene	Nasopharyngeal swab	40–60 min	Cleaves ssDNA. LED/UV-based detection.	[201]
